# A Subsampling Phase-Locked Loop with a Dual Charge Pump Based on Capacitor Multipliers for CMOS Image Sensor

**DOI:** 10.3390/mi16111266

**Published:** 2025-11-10

**Authors:** Yuguo Lin, Bin Wang, Liqing Jin, Ziyuan Tang, Fanshun Ye, Renjie Xie, Longsheng Wu, Guang Shi, Huan Liu

**Affiliations:** 1 State Key Discipline Laboratory of Wide Bandgap Semiconductor Technology, School of Microelectronics, Xidian University, Xi’an 710071, China; 2 School of Aerospace Science and Technology, Xidian University, Xi’an 710071, China

**Keywords:** sub-sampling phase lock loop, SSPLL, low jitter, capacitor multiplication, a dual charge pump

## Abstract

Traditional zero-compensation techniques employed to improve sub-sampling phase-locked loop (SSPLL) stability often exacerbate spur degradation or incur excessive area overhead, rendering them unsuitable for high-resolution image sensor applications. This paper proposes a novel SSPLL based on capacitor multiplication technology. This capacitor multiplication technology employs dual charge pumps (CP1 and CP2) in a coordinated operational scheme where their charge/discharge states are inversely synchronized. The effective capacitance of the loop filter is thereby amplified without expanding the physical layout area dedicated to capacitive components. Meanwhile, the continued use of zero-compensation technology ensures the stability of the SSPLL. The proposed SSPLL is designed and verified in a 55 nm CMOS process. At a 1.2 GHz output frequency, simulation results show a spot phase noise of −131.5 dBc/Hz at 1 MHz offset, accompanied by an integrated RMS jitter of 549 fs across the 10 kHz to 40 MHz spectrum, spurs suppressed to −51.3 dB, while maintaining a power efficiency of 3.81 mW and a compact layout area of 0.064 mm^2^. All the above results show that by introducing the novel dual-CP charge multiplication technology, the SSPLL can achieve low jitter and low power consumption performance while reducing the layout area, providing a new technical approach for its application in high-resolution image sensors.

## 1. Introduction

Image sensors are widely used in various fields, including infrared detection, autonomous driving, facial recognition, and machine vision [1]. High-order phase-locked loops (PLLs) serve as on-chip integrated clock signal generation circuits, providing reference clocks to sustain sensor operation [2]. Driven by the growing demand for high-resolution, high-frame-rate, high-dynamic-range, and compact-sized image sensors, the pixel pitch continues to shrink. This trend necessitates that product design prioritize area reduction as the primary objective, while minimizing clock jitter to the greatest extent, and power consumption constraints are becoming increasingly stringent.

The subsampling phase-locked loop (SSPLL) is widely used for its outstanding jitter performance, has grown in popularity for frequency synthesis [3]. Their sub-sampling phase detector (SSPD) can effectively suppress in-band noise [4,5,6], reduce dynamic spurs [7], and since the phase detection does not require the N-divider to operate, it avoids the issue in traditional phase-locked loops where noise factors are multiplied by the N2 factor [8,9]. During the locking process of the SSPLL, it adopts a structure that combines coarse adjustment with a high-current loop and fine adjustment with a low-current loop to enhance the locking speed. However, during the switching process between the two loops, due to different error detection methods and differences in loop gain, there can be insufficient phase margin and system stability issues, and it always leads to harmonic locking [10]. To enhance the system stability, loop filters require a low frequency zero to create a low bandwidth PLL for low in-band jitter performance, and it needs either a large capacitor or a large resistor. A large resistor can add thermal noise and increase the reference spurs of the PLL [11]. Thus, a large capacitor is preferred for low in-band noise performance, resulting in an increased area. As a viable solution, the technology of capacitor multiplication [12,13,14,15] is a key method for optimizing layout area. The authors in [16] used a three-stage current mirror for the proposed capacitor multipliers in SSPLL. By increasing the actual current flowing through the capacitor, the equivalent impedance transformation is achieved and capacitor area is reduced. However, due to the adoption of three-stage current mirror technology, the static power dissipation increases proportionally to the multiplication factor, reaching 8.42 mW. In addition, the mismatch between these transistors of the current mirror affects the accuracy of the multiplication factor. Therefore, this approach is unsuitable for applications requiring precise multiplication factors and poses challenges for deployment in ultra-large-scale image sensors with compact form factors and high-performance demands.

This paper introduces a novel SSPLL based on capacitor multiplication technology. Implemented with dual charge pumps, the core innovation of this technology lies in coordinating the complementary charge/discharge operations of the two charge pumps (CP1 and CP2), which operate in opposite states. By leveraging the effective capacitance of the multiplied loop filter, the layout area is significantly reduced while maintaining excellent jitter and spur performance, without degrading power consumption performance. Additionally, the use of zero-compensation technology ensures the stability of the SSPLL (the variation in phase margin is modeled and verified using MATLAB R2024a). The SSPLL, designed and verified in a 55 nm CMOS process, delivers output signals from 0.4 GHz to 2 GHz using a 40 MHz reference clock. At 1.2 GHz output frequency, simulation results demonstrate a spot phase noise of −131.5 dBc/Hz at 1 MHz offset, accompanied by an integrated RMS jitter of 549 fs across the 10 kHz to 40 MHz spectrum, spurs suppressed to −51.3 dB, while maintaining a power efficiency of 3.81 mW and a compact layout area of 0.064 mm^2^. The rest of this paper is organized as follows. Section 2 provides a comprehensive design and implementation architecture of various loop parameters. Additionally, we discuss the limitations of traditional architectures and introduce the concept of the dual charge pump, whose principles and implementation are shown in this section. Results and discussions are presented in Section 3. Finally, the conclusion is derived in Section 4.

## 2. Architecture and Design

**Figure 1:**
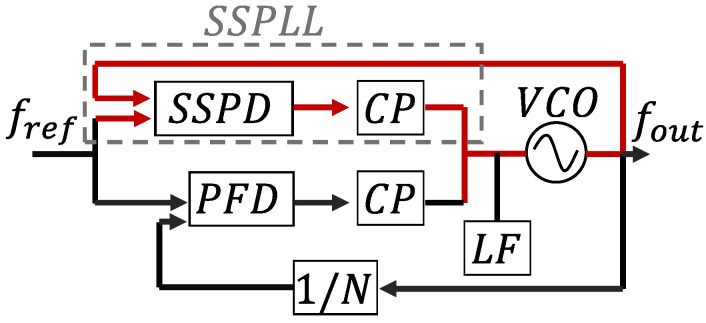
Block diagram of SSPLL.

**Figure 2:**
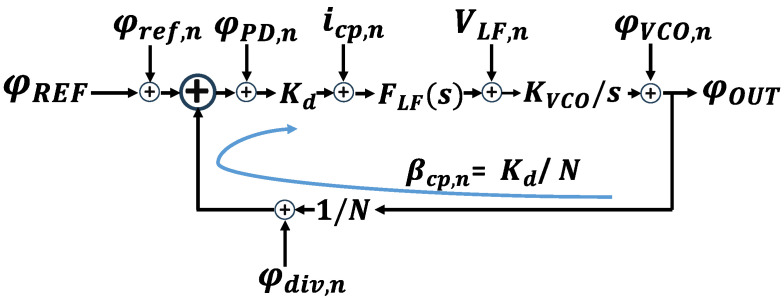
Phase domain model of classic PLL.

As shown Figure 1 and Figure 2 represent the SSPLL structure and the linear phase domain model of classic PLL.

By ignoring the flicker noise, we focus on the fundamental limitations due to thermal noise. Define the loop gain $\beta_{\mathrm{CP}}$ as $K_{d}/N$, then we can write the transfer function (where G(s) is the open-loop transfer function) as follows:

(1) $$H_{CP}\left(s\right)=\frac{1}{\beta_{CP}}\frac{\frac{\beta_{CP}F_{LF}\left(s\right)K_{VCO}}{s}}{1+\frac{\beta_{CP}F_{LF}\left(s\right)K_{VCO}}{s}}=\frac{1}{\beta_{CP}}\frac{G\left(s\right)}{1+G\left(s\right)}$$

Since noise passing through a linear time-invariant system is shaped by the transfer function to the original $H_{\mathrm{CP}}^{2}\left(s\right)$ times, its in-band noise (one-sided spectrum) can be written as follows:

(2) $$L_{in-band,CP}\approx \frac{S_{iCP,n}}{2\beta_{CP}^{2}}$$

Due to the 1/N factor in β, the noise is multiplied by $N^{2}$, the in-band output phase noise is as follows:

(3) $$L_{in-band,PLL}=\left(L_{REF}+L_{PFD,CP}\right)N^{2}$$

**Figure 3:**
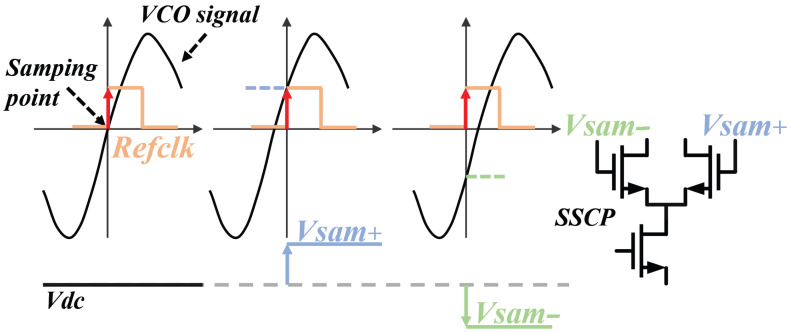
The sampling process of Sub-Sampling Phase Detector.

To mitigate this effect, a dual-loop structure was used in this instance. The conventional structure and phase detection method of the SSPLL are shown as follows:

From Figure 1, we can find that the SSPLL does not require the feedback of a frequency divider, directly comparing the high and low levels of the sampling voltage with the reference voltage. The sampling principle and signal transmission path are demonstrated in Figure 3, while the structural configuration of SSCP is illustrated in Figure 7. In comparison, it reduces the in-band noise as follows:

(4) $$L_{in-band,SSPLL}=L_{REF}N^{2}+L_{SSPD,CP}$$

Due to the significant low-frequency noise gain of the charge pump in the classical PLL, the in-band noise of the SSPLL is fully suppressed after the PLL loop is closed. To further improve noise performance and enhance the suppression of reference spurs, a third-order passive filter was chosen in this design. High-order filters can be broken down into a combination of low-order filters. To simplify the process, an analysis of the second-order filter can be conducted first, from which the transfer function can be derived as follows:

(5) $$Z_{LPF}\left(s\right)=\frac{1+sR_{1}C_{1}}{C_{1}C_{2}R_{1}s^{2}+\left(C_{1}+C_{2}\right)s}$$

from this, the zero $\omega_{Z}=\frac{1}{R_{1}C_{1}}$ and the pole $\omega_{P2}=\frac{C_{1}+C_{2}}{C_{1}C_{2}R_{1}}$ are calculated, along with the loop bandwidth $\omega_{C}=\frac{I_{CP}K_{VCO}R_{1}C_{1}}{2\pi N(C_{1}+C_{2})}$ and phase margin as follows:

(6) $$PM=\arctan\left(\frac{\omega_{C}}{\omega_{Z}}\right)-\arctan\left(\frac{\omega_{C}}{\omega_{p2}}\right)$$

When $C_{1}$ is much greater than $C_{2}$, the bandwidth can be approximated as $\omega_{C}\approx \frac{I_{\mathrm{CP}}K_{\mathrm{VCO}}V_{\mathrm{CO}}R_{1}}{2\pi N}$. After the loop switching to SSPLL, the $\omega_{C}$ can be expressed as follows:

(7) $$\omega_{C}=K_{SSPD/SSCP}K_{VCO}R_{1}$$

To ensure the phase margin of the PLL, the zeros of the loop gain should be positioned far from the loop bandwidth, and their ratio should remain fixed. To minimize jitter, the position of $\omega_{C}$ should be chosen to minimize the integral area of phase noise, at the intersection frequency of the VCO and charge pump phase noise output spectra [17]. A narrow bandwidth is a necessary condition to reduce spurs, but it may conflict with the optimal bandwidth for jitter performance, so they should be compromised [18]. Additionally, while the SSPLL effectively reduces in-band noise, the total phase noise is ultimately constrained by the maximum stable bandwidth of the phase-locked loop [19]. Therefore, leveraging the low-noise characteristics of the SSPLL, its loop bandwidth can be greater than that of the PLL loop. Since the loop gains are different, the bandwidths of the two loops cannot be consistent, so when it transitions from frequency locking to phase locking (which is when the classical PLL stops working), loop stability issues may arise. To measure the bandwidth between the two loops and compare them, a ratio is obtained.

(8) $$K_{\omega }=\frac{4\pi Ngm}{I_{CP}}\frac{T_{Pul}}{T_{REFCLK}}$$

**Figure 4:**
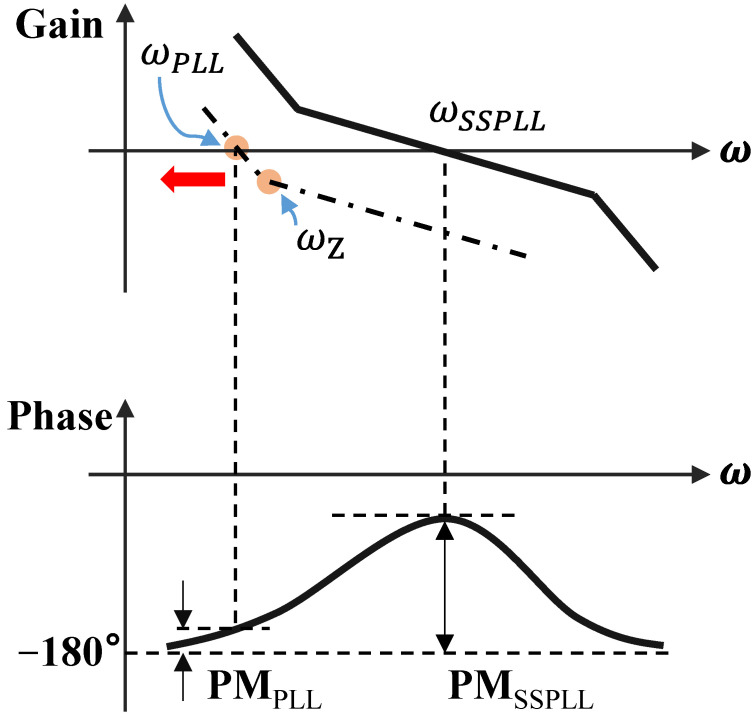
Phase margin (The red arrow indicates the direction of increasing N).

Especially when the division ratio *N* is high, it can be observed that its value is much greater than 1, including that the bandwidths between the loops differ significantly. In Equation (6), $\omega_{C}$ and $\omega_{Z}$ are too close to each other, leading to insufficient phase margin in the classical FLL loop. As shown in Figure 4, the gain curve of the FLL will shift to the left overall, a phenomenon that could potentially cause the loop to lock onto other harmonics or lose lock. To ensure the stability of the entire feedback system, the phase margin should be greater than $45^{\circ }$. The gain and phase curves are shown in the figures below. To address the stability issue, as referenced in Equation (8), one approach is to increase the charging and discharging current provided by the auxiliary frequency-locking loop charge pump; the other method is to maintain the ratio of $\omega_{C}$ to $\omega_{Z}$ without changing the pole position, which means reducing the value of the zero by increasing the value of $C_{1}$ in the loop filter. However, this method would significantly increase the area of passive components. Therefore, without changing the actual capacitance size, the goal can be achieved by increasing $C_{1}$ through capacitance multiplication technology.

**Figure 5:**
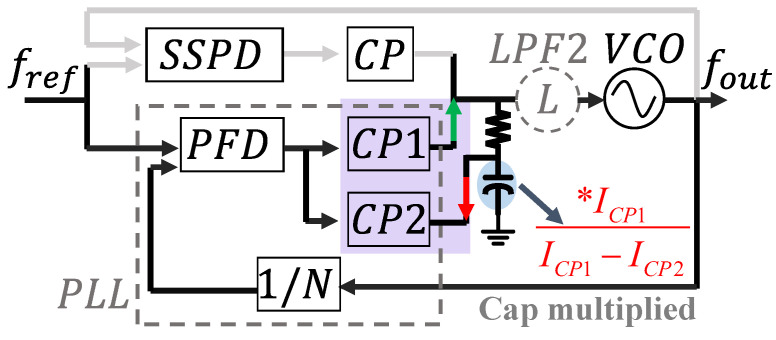
Synergistic capacitance multiplication mechanism in dual-charge-pump architectures.

This paper presents a design scheme for a dual charge pump implementation. As shown in Figure 5, implemented with dual charge pumps, the core innovation of this technology lies in coordinating the complementary charge/discharge operations of the two charge pumps (CP1 and CP2), which operate in opposite states. By leveraging the effective capacitance of the multiplied loop filter, the layout area is significantly reduced, without degrading power consumption and spur performance. The third-order filter is considered a parallel combination of a first-order filter and LPF2. The upper plate of the capacitor in the first stage is used as the node for the CP2 to draw current, with the charging current being M times the drawn current.

Since the junction of the second filter stage is a high impedance node, the voltage of the output would not be affected by drawn current, the key point is that the stabilizing jump on $R_{1}$ is still equal to $I_{1}R_{1}$. The behavior of the charge pump drawing current completes the impedance transformation of the entire loop filter and reduces the zero point to (M−1)/M times its original value.

(9) $$\frac{V_{\mathrm{cont}}}{\Delta \phi }\left(s\right)=\frac{Ip_{1}}{2\pi }\left(R_{1}+\frac{1}{C_{1}s}\right)-\frac{Ip_{2}}{2\pi }\frac{1}{C_{1}s}=\frac{Ip_{1}R_{1}}{2\pi }+\frac{Ip_{1}-Ip_{2}}{2\pi }\frac{1}{C_{1}s}$$

(10) $$\frac{V_{\mathrm{cont}}}{\Delta \phi }\left(s\right)=\frac{Ip_{1}R_{1}}{2\pi }+\frac{Ip_{1}}{2\pi }\frac{1}{\frac{C_{1}}{M-1}s}$$

**Figure 6:**
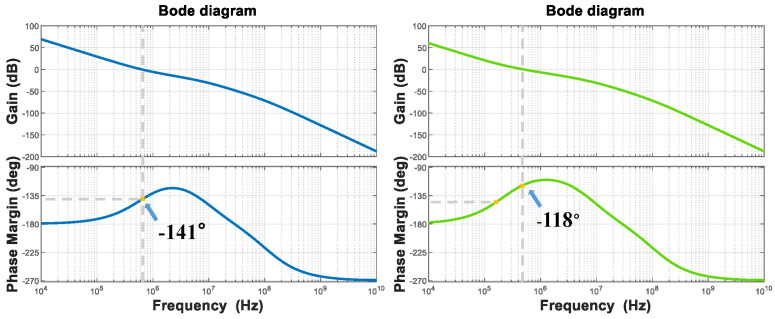
Bode plots before and after applying zero compensation technique.

Through modeling and verification in MATLAB, it can be intuitively observed from the Bode plots that the capacitance multiplication effect improves the phase margin. The comparison is shown in Figure 6.

### 2.1. The Design of a Dual Charge Pump

During the overall loop locking process, the classical PLL loop should play a key role in phase accumulation, so its charge pump charging and discharging current contributions should be significantly higher than those of the sub-sampling loop. In this design, the maximum current contributed by SSCP is 5 μA, hence 30 μA is chosen as the minimum charging and discharging current for the classical CP.

**Figure 7:**
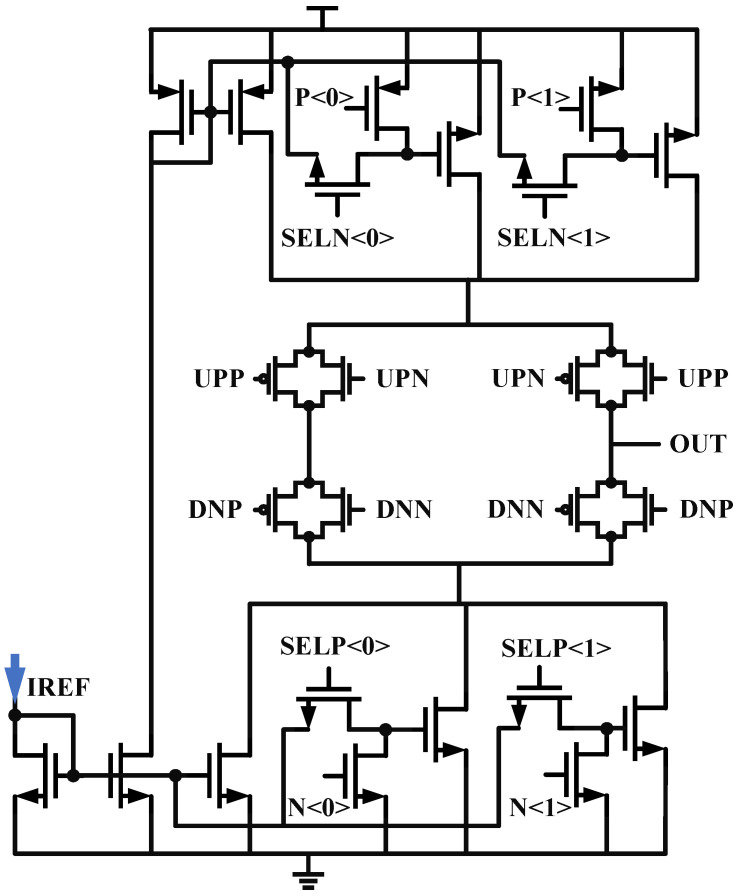
The schematic of Charge Pump.

By coordinating the two charge pumps, where CP1 charges and CP2 discharges, the first-level capacitor of the loop filter is doubled, becoming $\frac{I_{CP1}}{I_{CP1}-I_{CP2}}$. The pathway is labeled in Figure 5. The necessity of doubling and the issue of loop stability have been discussed in the previous text. To facilitate the adaptation of system lock time, CP1’s current is configured with a step size of 30 μA from 30 μA to 120 μA, and CP2’s current is configured with a step size of 20 μA from 20 μA to 80 μA. CP1 and CP2 share the same architecture, as shown in Figure 7, with a little difference in current configuration. To reduce leakage current, long-channel transistors are selected [20].

The input frequency of the phase-locked loop is 40 MHz; hence, a faster switching structure is required. Compared to source and drain control, gate control can provide an additional voltage margin of 2$I_{CP}R_{on}$. The switch resistances for NMOS and PMOS can be approximated as follows:

(11) $$R_{N}=\frac{1}{K_{N}{\left(\frac{W}{L}\right)}_{N}\left(V_{DD}-V_{in}-V_{thN}\right)}$$

(12) $$R_{N}=\frac{1}{K_{P}{\left(\frac{W}{L}\right)}_{P}\left(V_{in}-\left|V_{thP}\right|\right)}$$

Under the condition of the same switch resistance, using gate-controlled switches MOSFETs can significantly reduce the layout area, hence this design chooses them.

### 2.2. A Sub-Sampling Charge Pump

**Figure 8:**
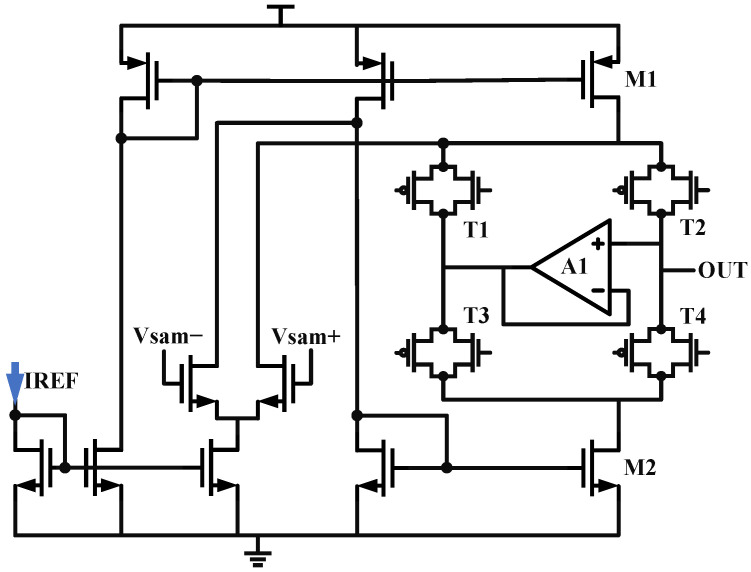
The schematic of Sub-Sampling Charge Pump.

For the classic charge pump structure, we first need to address the charge sharing issue: if M1 and M2 are only coupled to the output through ordinary switches, when the switches are turned off, their drain ends will be charged and discharged. When the switches are closed again, charge redistribution occurs between the capacitors. To suppress this non-ideal effect, this paper adds two switches, T1/T3, and a unity gain amplifier between the nodes where charge sharing occurs, proposing a new sub-sampling charge pump structure. The schematic is shown in Figure 8. When T2/T4 are turned off, T1/T3 are closed, and the clamping action of the op-amp makes the drain voltages of M1 and M2 the same as the output node voltage. When T2/T4 are turned on again, there is no potential difference, so the parasitic capacitance does not participate in the charge redistribution.

### 2.3. Rail-to-Rail Amplifier

To accurately implement the clamping function, an operational amplifier was designed in this paper. Due to the high requirement for swing amplitude, a rail-to-rail structure was chosen to increase the input voltage range. The low-frequency open-loop DC gain of the rail-to-rail amplifier is:

(13) $$\left|A_{open}\right|=gm_{17}\left(gm_{1}+gm_{3}\right)(r_{o17}\left|\right|r_{o18})*\left(gm_{15}r_{015}r_{o16}\left|\right|gm_{13}r_{013}r_{o14}\right)$$

The input is composed of a parallel connection of NMOS and PMOS differential pairs, and it is necessary to consider the variation in the upper and lower limits of the input transistor’s transconductance under the same current condition as follows:

(14) $$gm_{MAX}=\sqrt{2I_{N}K_{N}{\left(W/L\right)}_{N}}+\sqrt{2I_{P}K_{P}{\left(W/L\right)}_{P}}$$

(15) $$gm_{MIN}=\sqrt{2I_{N}K_{N}{\left(\frac{W}{L}\right)}_{N}}$$

In the equation, it can be observed that the maximum transconductance is twice the minimum value, resulting in a large variation range for the gain-bandwidth product, which is not conducive to frequency compensation.

**Figure 9:**
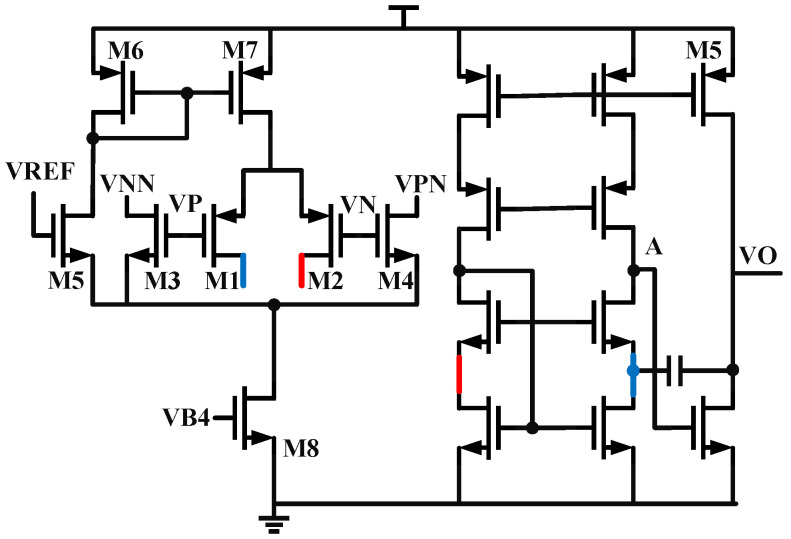
Rail-to-rail Amplifier.

As shown in Figure 9, the M1 M8 within the structure form the constant transconductance stage, and M1 M5 operate in the subthreshold region, with their transconductance given by the following:

(16) $$gm=\frac{qI}{mkT}$$

where the value of gm is only related to the current and temperature. When the input common-mode voltage is less than VREF, M5 turns on, and M3 and M4 turn off, with all the current passing through M5 M7, at which point only the PMOS differential pair operates; when the input common-mode voltage equals VREF, M3 M5 turn on simultaneously, with the NMOS and PMOS differential pairs each receiving half of the current, and due to the linear relationship between subthreshold transconductance and current, a constant transconductance input is achieved.

### 2.4. PFD with Additional Dead Zone

The presence of a dead zone can cause the phase-frequency detector to fail to precisely handle very small phase errors, as the output pulses generated by comparison are too narrow to effectively drive the charge pump, ultimately reducing the loop gain of the phase-locked loop and increasing the jitter. In other words, only when the phase difference between the reference signal and the feedback signal continuously accumulates to a range that the phase-frequency detector can discern, can it detect and drive the entire loop to form feedback. This can cause the phase-locked loop to never stabilize at the correct equilibrium point, with phase errors accumulating, and the VCO oscillating at the edges of the dead zone. In the frequency domain, this manifests as phase noise, and in the time domain, it appears as jitter in the output clock signal.

**Figure 10:**
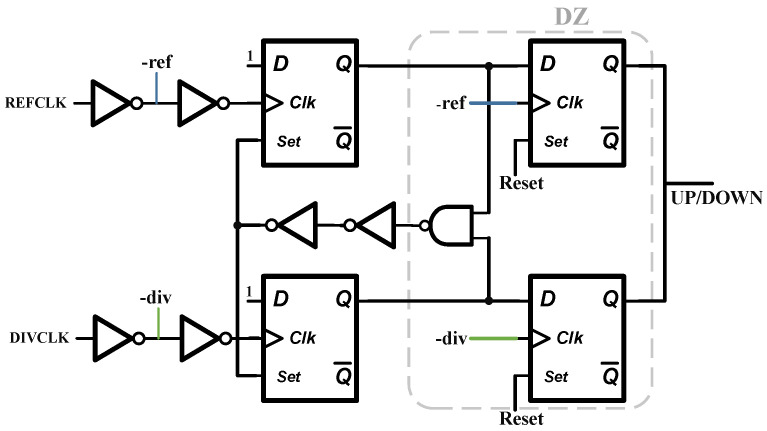
PFD-DZ.

For sub-sampling phase-locked loops, unlike the dead-zone elimination techniques used in traditional phase-locked loops, their phase-frequency detectors (PFDs) need to expand the range of the dead zone to quickly switch from the traditional loop to the sub-sampling loop. Thereby eliminating the contribution of traditional charge pumps and frequency dividers on in-band noise, and consequently reducing the power consumption associated with this part. This design employs a general-purpose sub-sampling phase detector architecture without extra customization, as shown in Figure 10, where the functional module generated by the dead zone is positioned on the right side of the circuit. By inverting the clock and inputting it into a D flip-flop, the dead zone is extended to the following:

(17) $$\varphi_{DZ}=\frac{2\pi \left(T_{REFHigh}+T_{DIVHigh}\right)}{T_{REFCLK}}$$

The equation shows that when the time difference between the rising edge of the REF signal and the DIV signal is within the high-level interval of the REF signal, the UPP signal controlling the charge pump does not respond. On the contrary, it outputs a high level to promote frequency catching up, and the work of catching up in phase is handed over to the sub-sampling loop to complete.

### 2.5. Voltage-Controlled Oscillators

**Figure 11:**
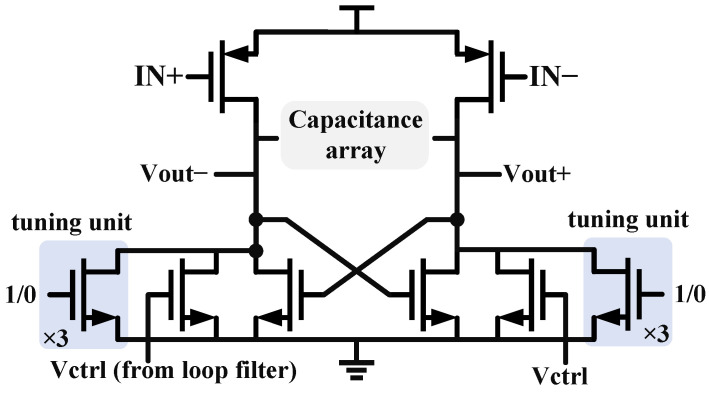
Delay unit of Ring VCO.

The structure of voltage-controlled oscillators can generally be divided into two types: Capacitor–Inductor oscillators (LC VCO) and ring oscillators (ring VCO). The LC type VCO has low output phase noise but requires a larger layout area. Therefore, considering the application background of a small area for image sensors, a three-stage ring oscillator structure is chosen for this design. Figure 11 shows the unit structure. The classic variable resistor delay cell and three pairs of controllable tuning switches have been added in parallel on the original basis to cope with a wider range of output frequencies. And the controllable capacitor array can further refine the tuning range and fill the gaps between tuning steps. Cross-coupled NMOS topology structure can provide a larger margin of variation and low parasitic capacitance [21].

Relaxation oscillators have a profound scope as on-chip reference clock sources or sensor front-ends in comparison to ring oscillators due to their superior frequency stability, control linearity, and wide tuning range. However, their phase noise performance lags behind that of ring oscillators. Relaxation oscillators are primarily used to generate clock signals in the MHz range (probably 10 MHz) [22,23]. LC oscillators exhibit less phase noise than ring oscillators (for the same power consumption), higher oscillation frequencies, and voltage excursions beyond the supply rails. The disadvantages are a narrower tuning range and significant area consumption. Therefore, based on the design specifications of this work, a ring oscillator is a more reasonable choice [24,25].

## 3. Simulation and Results

**Figure 12:**
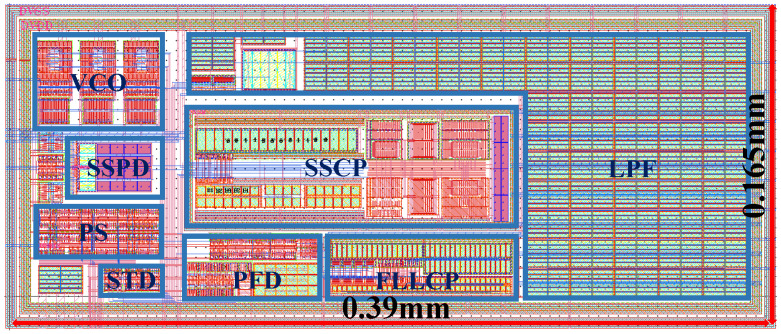
Layout.

After completing the design and verification of each module, we designed and tested the layout under 300 K temperature and the tt corner. As shown in Figure 12, guard rings have been added around the outer perimeters of each functional module to provide isolation. Aiming to minimize the area, this design ensures that the layout of each module is as compact as possible.

**Figure 13:**
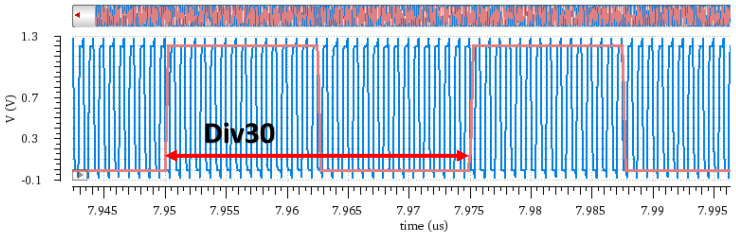
Output clock wave and input wave.

The test conditions are an input frequency of 40 MHz, division ratio set to 30, dual charge pump currents of 90 μA and 60 μA, and the following results were obtained at 300 K temperature and under the typical process corner. Figure 13 shows the output signal and input signal waveform; a signal with an output frequency of 1.2 GHz is generated, thus fulfilling the design functionality.

**Figure 14:**
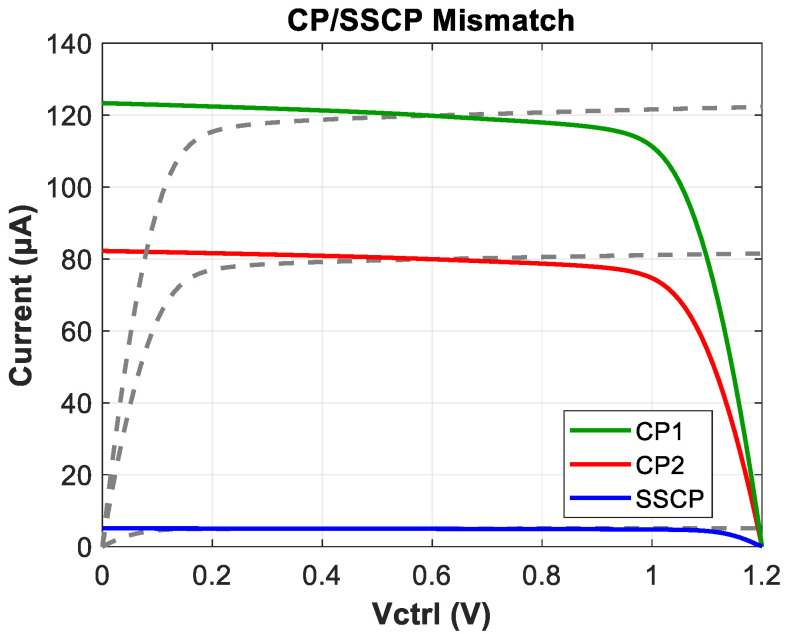
Mismatch of CP and SSCP.

Figure 14 shows the mismatch of charge pumps. We do not overly emphasize the precision of capacitance multiplication but focus on ensuring the phase margin remains within a safe operating range. The overall mismatch ratio of the two CPs under normal operating voltages is calculated as 1.21%, slightly higher than that of the SSCP (0.62%).

**Figure 15:**
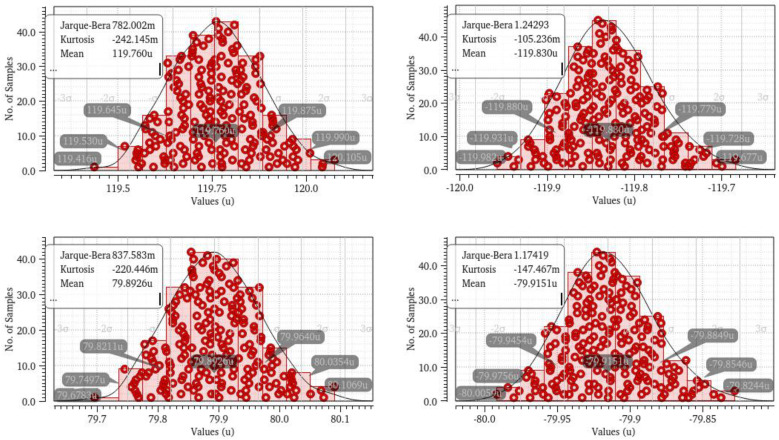
Monte Carlo simulations of charge pumps.

Figure 15 shows the Monte Carlo simulations of charge pumps, The worst-case scenario of charge pump mismatch (1.56%) results in certain deterioration of phase noise and spur performance, but this impact is acceptable.

**Figure 16:**
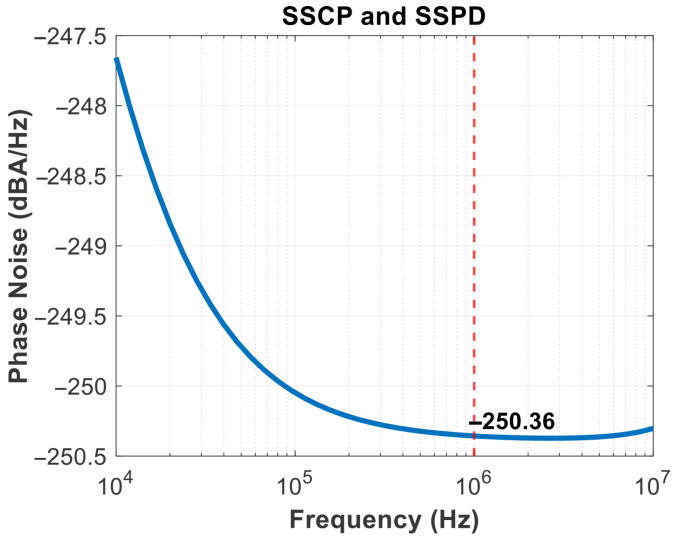
SSPD and SSCP-cascaded noise.

**Figure 17:**
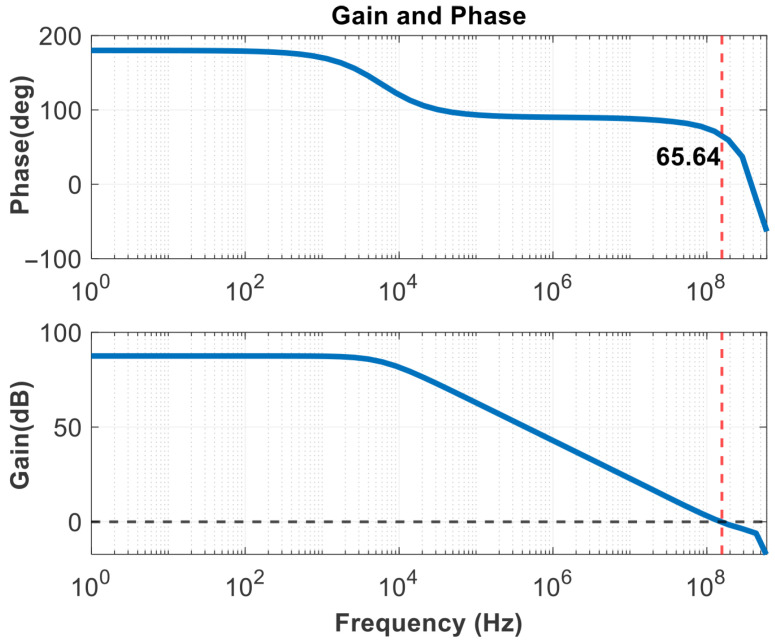
Amplitude and phase frequency characteristic.

**Figure 18:**
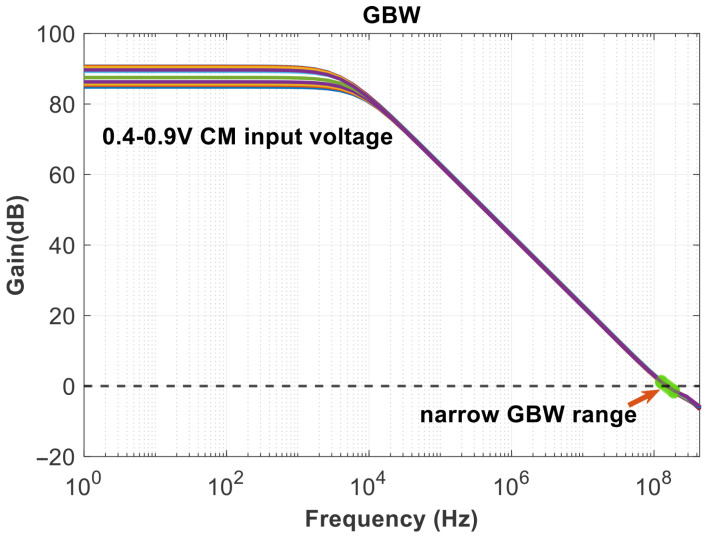
GBW in different common mode voltage.

The SSPD and SSCP are cascaded to obtain the noise results in Figure 16, in-band noise is primarily generated by their outputs. It shows that the current noise reaches −250 dBA/Hz at a frequency offset of 1 MHz. And the linearity of the SSPD/CP is a matter of concern, as it affects the stability of the loop gain [26]. Therefore, it is also possible to consider transforming the sine wave into a more linear waveform, such as a sawtooth wave, before operating. As the operating temperature rises, the device’s thermal noise increases significantly, charge pump mismatch and leakage worsen, affecting the ripple of $V_{c}trl$ and leading to degradation in spur and jitter performance. The gain and phase results of the rail-to-rail amplifier are shown in Figure 17. Figure 18 illustrates the GBW stability of this amplifier. From the scanning results below, it can be obtained that the minimum value is 147.729 MHz and the maximum value is 161.732 MHz under different common-mode voltage levels. The range of variation is small, and the error is within the allowable design range, indicating that the input transconductance is relatively constant and does not affect the rapid implementation of the clamping function.

**Figure 19:**
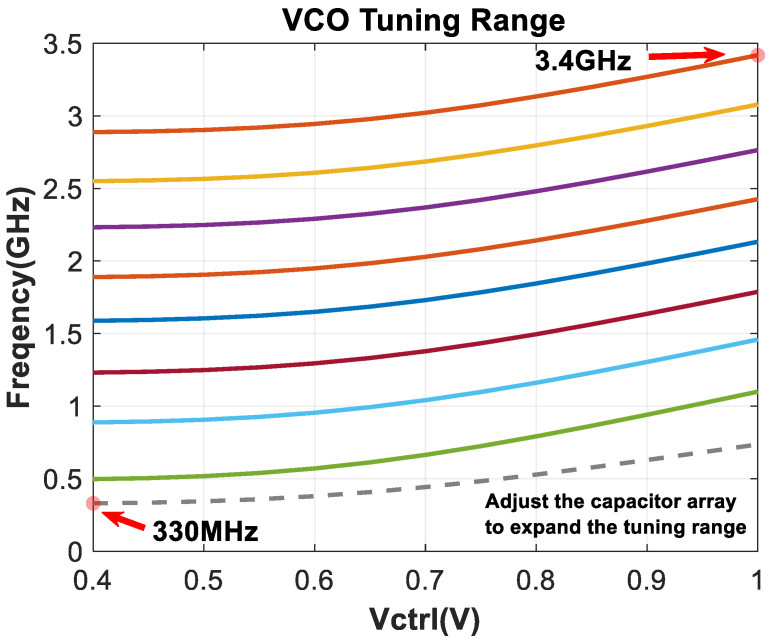
VCO tuning range.

Figure 19 shows the VCO tuning range, which arises from the unit structure detailed in Figure 11. The eight solid lines represent adjustable frequency levels controlled by MOS transistors, while the gray dashed line at the bottom denotes frequency fine-tuning via a capacitor array, specifically designed to cover the minimal resonant frequency points. This configuration comprehensively addresses the division ratio requirements of the frequency divider; the output frequency range is 0.33–3.4 GHz under the conditions of a 300 K temperature and the tt process corner.

**Figure 20:**
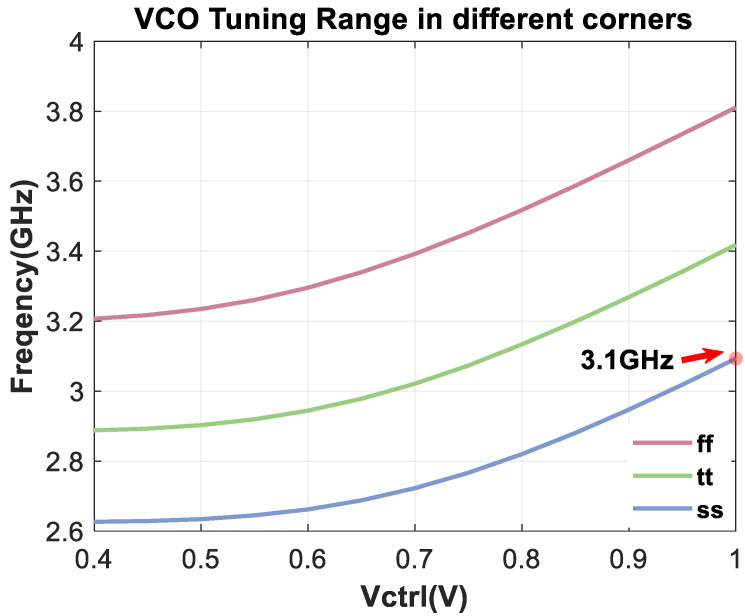
VCO’s maximum tuning ranges in different corners.

The variations in the VCO’s maximum tuning ranges under different process corners are presented in Figure 20. The results indicate that even with the minimal tuning range under the ss corner (the minimum output frequency of corners), the design specifications can still be satisfied.

**Figure 21:**
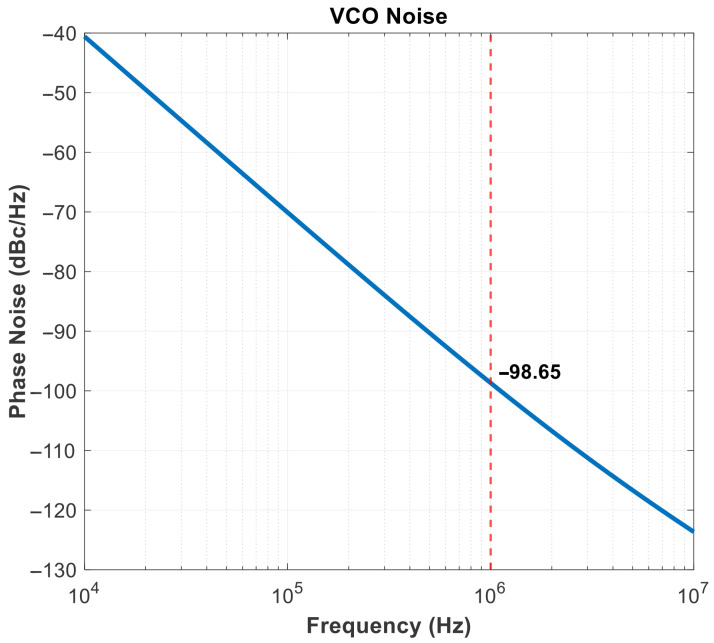
VCO Pnoise.

Figure 21 shows the phase noise of VCO, which is the most important part of RMS jitter. When the voltage-controlled oscillator is operating at an output frequency of 1.2 GHz, the output phase noise is −98.65 dBc/Hz, indicating excellent noise performance.

**Figure 22:**
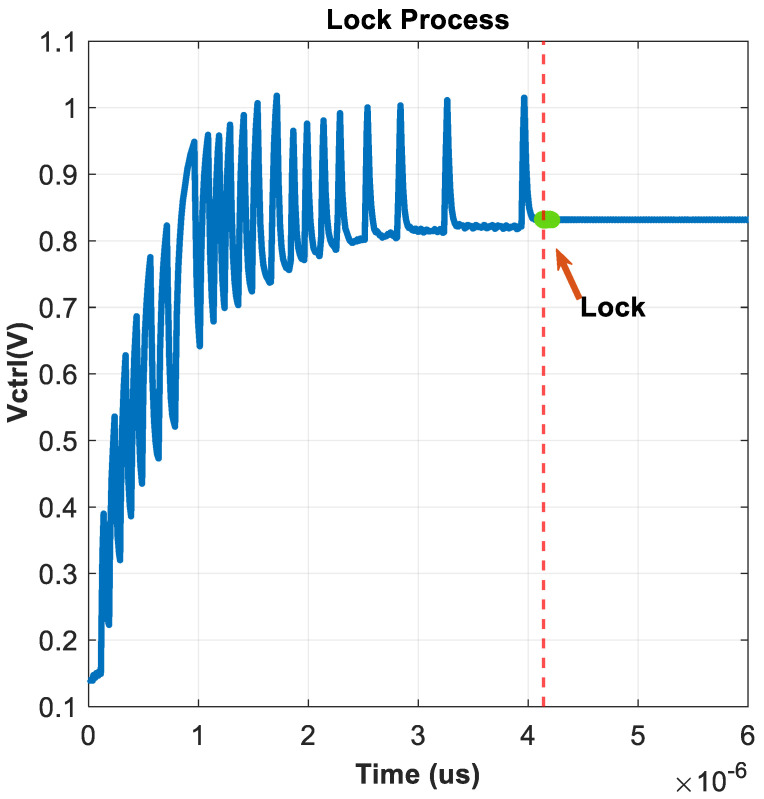
Lock time of SSPLL.

**Figure 23:**
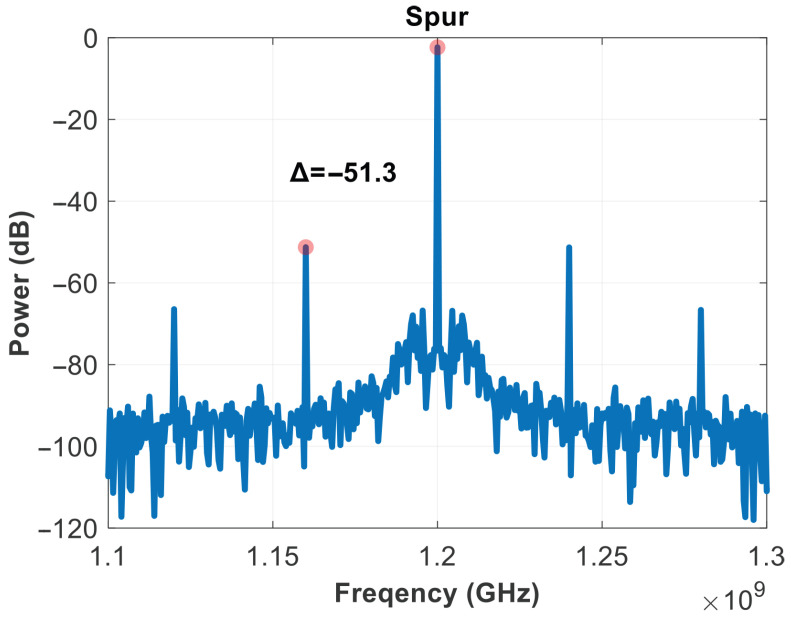
Spur of output.

**Figure 24:**
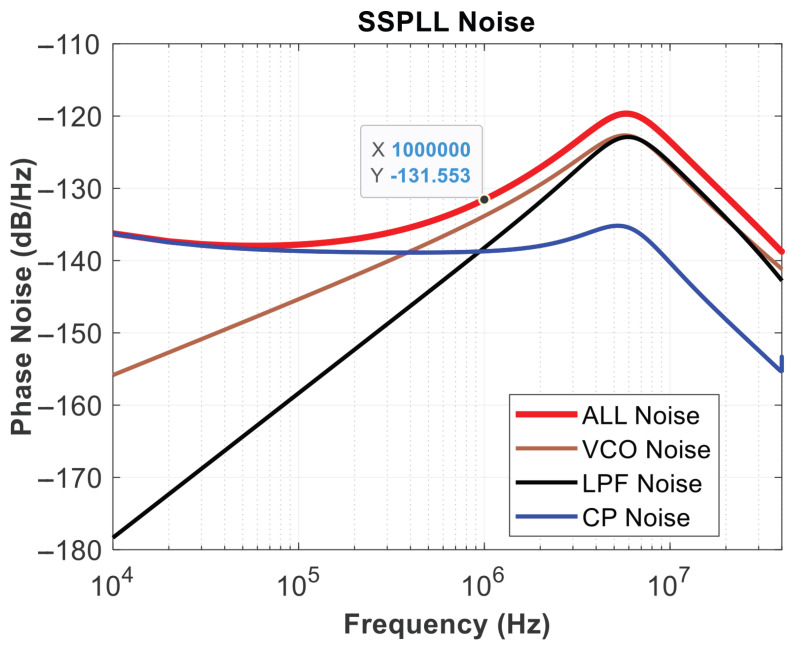
RMS jitter.

The performance of the system can be evaluated in terms of lock time in Figure 22, spur in Figure 23, and RMS jitter in Figure 24.

The SSPLL can lock in a short time, about 4us from Figure 22. The occurrence of voltage spikes is primarily due to the charging current of the PLL loop, which is used to quickly catch up with the phase. After the loop has locked, loop power consumption is 3.81 mW, and spurs are −51.3 dB from Figure 23. Based on the comparison in Table 1, this is clearly a good performance indicator. The RMS jitter was fitted using MATLAB, depicting the simulated phase noise contribution of different SSPLL blocks, yielding a jitter of 549 fs and an overall output noise of −131.5 dBc/Hz at 1 MHz in Figure 24.

By comparing the work of this paper with the indicators in the recent literature in Table 1, there has been a significant optimization in terms of area; it is the key benefit of a dual charge pump technology.

## 4. Conclusions

This paper introduces a low jitter SSPLL with a dual charge pump, implemented in an icdr 55 nm CMOS process. The major aspect of analysis was phase noise and the use of capacitance multiplication technology. The design of dual charge pumps enables capacitance multiplication within the loop filter, enhances phase margin by optimizing charge transfer dynamics, and effectively mitigates system instability. Tests show that the SSPLL achieves an output signal frequency range of 0.4 GHz to 2.4 GHz. At a power supply voltage of 1.2 V and an output frequency of 1.2 GHz, the following performance is achieved: a total current consumption of 3.177 mA, clock RMS jitter of 549 fs, spurs of −51.3 dB, and a layout area of 0.064 mm^2^. Low phase noise, low power consumption, and compact area will continue to drive PLL research.

## Figures and Tables

**Table 1:** Performance comparison with other related works.

Category	JSSC2009 [9]	JSSC2021 [27]	JSSC2020 [28]	JSSC2020 [29]	This Work
**Process (nm)**	65	130	65	65	55
**Architecture**	Analog SSPLL	Analog SSPLL	Analog SSPLL	Analog SSPLL	Analog SSPLL
**$f_{OUT}$ (GHz)/$f_{REF}$ (MHz)**	2.21/55.25	2.3/50	2.4/100	6.8/106.25	0.4-2.4/40
**Dividing ratio**	40 (Integer-N)	64 (Integer-N)	24 (Integer-N)	64 (Integer-N)	60 (Integer-N)
**Reference spur (dBc)@ $f_{OUT}$ (GHz)**	−46	NA	−67	−40	−51
**Total power (PDC) (mW)**	7.6	2.8	0.93	2.3	3.81
**Active area** (mm^2^)	0.18	0.4	0.24	0.25	**0.064**

## Data Availability

The original contributions presented in this study are included in the article. Further inquiries can be directed to the corresponding author.
